# A Comprehensive Review of Performance Metrics for Computer-Aided Detection Systems

**DOI:** 10.3390/bioengineering11111165

**Published:** 2024-11-19

**Authors:** Doohyun Park

**Affiliations:** VUNO Inc., Seoul 06541, Republic of Korea; dhpark.ee@gmail.com

**Keywords:** performance metric, computer-aided detection, receiver operating characteristic, free-response receiver operating characteristic, alternative free-response receiver operating characteristic, artificial intelligence, lung nodule

## Abstract

This paper aims to provide a structured analysis of the performance metrics used in computer-aided detection (CAD) systems, specifically focusing on lung nodule detection in computed tomography (CT) images. By examining key metrics along with their respective strengths and limitations, this study offers guidelines to assist in selecting appropriate metrics. Evaluation methods for CAD systems for lung nodule detection are primarily categorized into per-scan and per-nodule approaches. For per-scan analysis, a key metric is the area under the receiver operating characteristic (ROC) curve (AUROC), which evaluates the ability of the system to distinguish between scans with and without nodules. For per-nodule analysis, the nodule-level sensitivity at fixed false positives per scan is often used, supplemented by the free-response receiver operating characteristic (FROC) curve and the competition performance metric (CPM). However, the CPM does not provide normalized scores because it theoretically ranges from zero to infinity and largely varies depending on the characteristics of the data. To address the advantages and limitations of ROC and FROC curves, an alternative FROC (AFROC) was introduced to combine the strengths of both per-scan and per-nodule analyses. This paper discusses the principles of each metric and their relative strengths, providing insights into their clinical implications and practical utility.

## 1. Introduction

Recent advances in artificial intelligence (AI) technology have been applied in various domains, particularly in medical imaging [1,2,3]. AI-based computer-aided detection (CAD) systems have become indispensable tools in modern radiology, pathology, and other medical fields. These systems facilitate faster and more accurate screening and diagnosis while also reducing costs [4,5].

One of the most promising applications of CAD systems is the early detection of lung cancer using chest computed tomography (CT). Early detection enables timely intervention in patients with lung cancer, reducing the likelihood of tumor growth or metastasis and increasing survival rates [6,7,8,9]. However, even board-certified radiologists often miss nodules, and there is significant inter-reader variability [10]. Consequently, the demand for automated CAD systems is growing. Numerous reader studies have shown that the use of CAD systems improves diagnostic performance, not only for junior radiologists, but also for experienced radiologists with more than 10 years of practice [11,12]. Moreover, the effectiveness of CAD systems continues to be demonstrated in stand-alone studies that evaluate these systems independently [13]. However, there is no single gold-standard metric to evaluate the performance of these systems; each metric has its own strengths and limitations [14]. In this study, we introduce various evaluation metrics, explain how each can be interpreted, and identify the specific scenarios in which they are most appropriate.

In this study, lung nodule detection in CT scans was used as a representative application to explain the evaluation metrics of the CAD systems. The LUng Nodule Analysis 2016 (LUNA16) challenge has played a key role in establishing standards for lung nodule detection [15]. In LUNA16, the performance of algorithms is mainly evaluated using a free-response receiver operating characteristic (FROC) curve, which plots sensitivity against the average number of false positives (FPs) per scan. The competition performance metric (CPM), an overall sensitivity score averaged at predefined FP thresholds, further facilitates comparison by summarizing performance across multiple FROC points [16]. In addition, the LUNA16 challenge provides an open-source benchmark dataset with annotated center points and diameters for each nodule, enabling researchers worldwide to evaluate the detection model performance using a common dataset. Rather than using the commonly employed average precision (AP) based on intersection over union (IoU) in the computer vision field, LUNA16 uses a matching criterion in which a predicted object is considered to match a ground truth nodule if the distance between its center points is smaller than the nodule radius. Nonetheless, FROC curve analysis and the CPM have clear limitations; therefore, in many cases, other metrics should be provided to complement the analysis of system performance.

For example, Baumgartner et al. [17] introduced nnDetection, a publicly available tool that automates the configurations required for model training based on the dataset used. They demonstrated nnDetection’s performance in various tasks, including the LUNA16 challenge dataset. Using FROC curves and CPM scores, they highlighted their superiority over other methods. Additionally, NVIDIA’s open-source framework, the Medical Open Network for Artificial Intelligence (MONAI), provides a pretrained detection network for lung nodule detection [18]. It includes FROC results and additional metrics such as the mean AP (mAP) and mean average recall (mAR). Many other studies have also used the LUNA16 challenge dataset but reported not only the FROC and CPM but also accuracy, sensitivity, and specificity [19]. Although the FROC shows results at specific FPs per scan, standardization is lacking, as each study used different thresholds such as 1.057, 2.1, 3.8 or 15.28 FPs per scan [20]. Thus, the evaluation of CAD systems for lung nodule detection faces the issue of a lack of standardized criteria.

Although standardized criteria are lacking, performance metrics for lung nodule detection can be categorized into per-scan and per-nodule analysis. The per-scan analysis focuses on the ability of the system to correctly identify scans with and without nodules. The area under the receiver operating characteristic (ROC) curve (AUROC) is commonly used for this purpose. It requires data from scans with and without nodules to assess the ability of the system to discriminate between the two. However, it was not possible to evaluate each individual nodule in the scan.

Per-nodule analysis provides a more detailed assessment by evaluating the ability of the CAD system to detect individual nodules within the scans. For this purpose, the FROC curve and CPM are commonly used. However, the CPM is not a standardized metric, as the horizontal axis can range from zero to infinity depending on the characteristics of the data. Consequently, researchers have not proposed an area under the FROC curve. Additionally, detecting more true nodules with a fixed positive predictive value (PPV) may result in a higher number of FPs per scan, potentially skewing the FROC curve and misrepresenting the model’s detection performance [21].

Both ROC and FROC curves, along with their respective quantitative metrics, the AUROC and CPM, have advantages and disadvantages. To complement these metrics, an alternative FROC (AFROC) metric was introduced [14]. The AFROC combines both per-scan and per-nodule analyses, plotting specificity from a per-scan perspective and sensitivity from a per-nodule perspective. Similarly to the AUROC, the area under the AFROC curve (AUAFROC) provides a normalized value between zero and one.

This study provides a comprehensive overview of the performance metrics for evaluating CAD systems. For each metric, we discuss what it measures and how it can be interpreted in clinical contexts with a specific focus on lung nodule detection.

## 2. Quantification Methods

**Figure 1:**
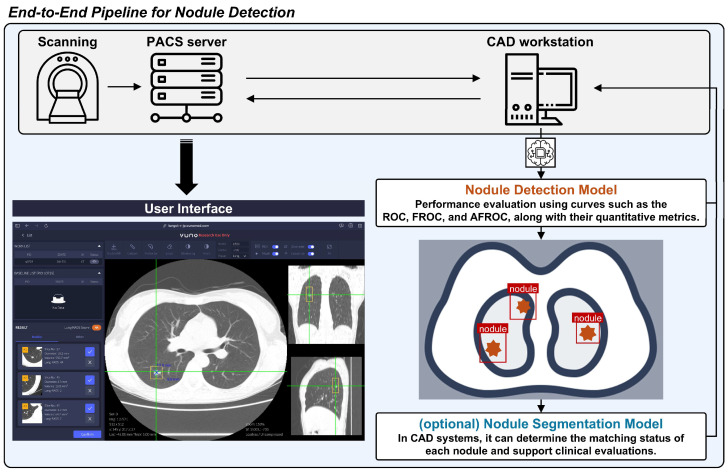
A pipeline of the computer-aided detection (CAD) system for lung nodule detection in computed tomography scans. PACS, picture archiving communication system; ROC, receiver operating characteristics; FROC, free-response ROC; AFROC, alternative FROC.

The general pipeline of the CAD system for lung nodule detection on CT scans is shown in Figure 1. The evaluation of CAD systems for lung nodule detection in chest CT scans is broadly divided into per-scan and per-nodule analysis. This section provides an explanation of each as well as a combined analysis method that incorporates both per-scan and per-nodule approaches. Per-scan analysis should be understood as evaluating normal and abnormal scans separately, with the overall assessment being a combination of both. The per-nodule analysis approach differs somewhat from per-scan analysis. Per-nodule analysis evaluates the performance based on all detected nodule candidates, regardless of whether a scan contains nodules.

In the field of computer vision, metrics such as the precision–recall (PR) curve and AP are widely used because they focus on the accuracy of positive predictions. However, in clinical diagnostics, in which distinguishing between disease and non-disease cases is essential, the ROC curve is generally preferred. Although PR curves effectively emphasize true positive detection, they do not account for true negatives, which limits their utility in comprehensive clinical evaluations. Therefore, this study used the ROC, FROC, and AFROC curves, as they align better with the clinical goals of lung nodule detection in chest CT scans.

### 2.1. Per-Scan Analysis

#### 2.1.1. Normal Scan Evaluation

Consider normal scan A without nodules processed by the CAD system. The output consists of bounding boxes around non-nodule objects. Each bounding box is assigned a confidence value that represents the probability that the object within it is a nodule. If this probability exceeds a predefined threshold set by the CAD system, then the object within the bounding box is classified as an FP nodule.

In per-scan analysis, the likelihood that normal scan A contains a nodule is determined by the maximum confidence value among all FPs, as shown in Equation (1), where *p* represents the probability that the object within the bounding box is a nodule, and *N* represents the number of FPs.

(1) $$\mathrm{Normal}\;\mathrm{Scan}\;\mathrm{Probability}=\max\left\{p_{N}\right\}$$

**Figure 2:**
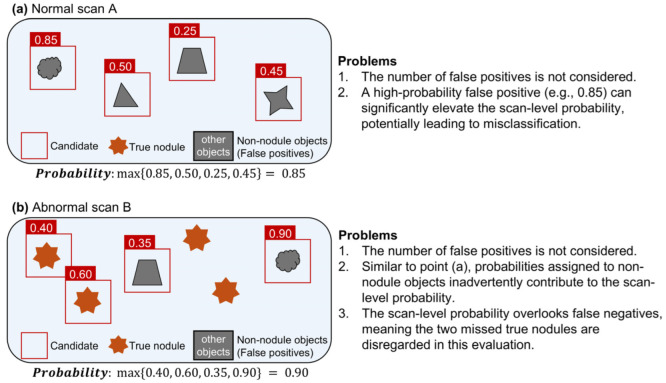
Examples illustrating issues with scan-level probability in computer-aided detection. (**a**) A case with normal scan not including nodules. (**b**) A case with abnormal scan including nodules.

In this analysis, only the FP with the highest probability is used, regardless of the total number of FPs (Figure 2a). In a deep learning-based CAD system, if no bounding box exceeds the decision threshold, the scan is considered to have no FPs, and the probability of a nodule being present in that scan is set to 0.

However, to accurately determine the decision threshold for nodule detection using a CAD system, it is essential to evaluate abnormal scans. Without incorporating abnormal scans, setting the decision threshold at one would yield a negative predictive value (NPV) of one but result in a PPV of zero.

#### 2.1.2. Abnormal Scan Evaluation

Consider an abnormal scan, B, that contains one or more nodules and is processed using the CAD system. The output consists of bounding boxes around the detected objects, with each bounding box assigned a confidence value. If this probability exceeds a predefined threshold set by the CAD system, the object within the bounding box is considered a nodule candidate. A nodule candidate can be a true positive (TP) if it is indeed a nodule or an FP if it is not.

In per-scan analysis, the likelihood of the presence of a nodule in abnormal scan B is assessed at the scan level. The probability of abnormal scan B is defined as the maximum confidence value among all the detected objects, as shown in Equation (2), where *p* represents the confidence that a bounding box contains a nodule, and *M* denotes the number of detected objects.

(2) $$\mathrm{Abnormal}\;\mathrm{Scan}\;\mathrm{Probability}=\max\left\{p_{M}\right\}$$

In this analysis, the numbers of TPs and FPs are not considered. Instead, the focus is on a single detected object with the highest probability, regardless of the total number of detected objects. Because the number of FPs is not considered in the evaluation of normal scans, similar issues arise when evaluating abnormal scans.

**Problem** **1.** *The scan-level probability for an abnormal scan does not differentiate whether the highest probability is associated with a TP or an FP. Furthermore, it does not account for the number of false negatives (FNs). As illustrated in Figure 2b, suppose abnormal scan B contains four true nodules and yields four candidate objects with probabilities of 0.35, 0.40, 0.60 and 0.90, respectively. If the actual nodule statuses for these objects are non-nodule, nodule, nodule, and non-nodule, the scan-level probability is 0.9. This occurs even though the highest probability is associated with a non-nodule, which indicates an FP. Furthermore, the actual sensitivity of scan B is 0.5. However, scan-level evaluations do not account for such factors, which can distort the results.*

#### 2.1.3. Quantification

**Figure 3:**
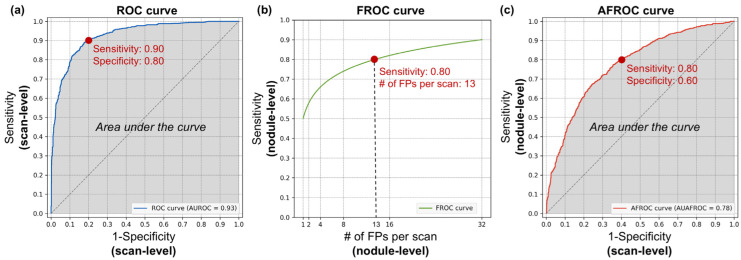
Example curves for evaluating computer-aided detection systems. The closer each curve is to the top left corner, the better the performance. (**a**) Receiver operating characteristic (ROC) curve and the area under the ROC curve (AUROC). (**b**) Free-response ROC (FROC) curve. (**c**) Alternative FROC (AFROC) curve and the area under the AFROC curve (AUAFROC). In each curve, the red dot indicates the model’s performance at a specific example cut-off threshold. FP, false positive.

The ROC curve is an evaluation method that comprehensively represents performance across both normal and abnormal scans. The horizontal axis corresponds to the FP rate, indicating the performance on normal scans, and is equivalent to 1-specificity. The vertical axis represents the TP rate, which reflects the performance on abnormal scans and is equivalent to sensitivity. A curve closer to the upper left corner indicates a better performing model. An example of an ROC curve is shown in Figure 3a. The area under the ROC curve is referred to as the area under the curve (AUC) or AUROC. Although the term AUC can be ambiguous without specifying the type of curve, it is widely used to denote the area under the ROC curve unless stated otherwise. This value serves as a quantitative measure of performance, ranging from zero to one, with higher values indicating a better diagnostic ability. However, if the model makes random guesses, the graph will theoretically approach the line y = x. In this case, the AUC becomes 0.5, so the practical range of AUC values is from 0.5 to 1.

Per-scan analysis is an effective metric for evaluating the performance of CAD systems for both normal and abnormal scans. However, as mentioned previously, this method has limitations. Because the scan-level probability is defined as $\max\left\{p_{N}\right\}$, it does not provide information on the number of FNs and FPs generated by the CAD system.

#### 2.1.4. Clinical Implication

The ROC curve and AUROC values did not evaluate each detected object at the nodule level, making them more suitable for assessing the screening performance of a CAD system at the scan level. Specifically, the specificity of the ROC curve for normal scans helps to determine the number of scans that can be excluded from further evaluation. Conversely, the sensitivity or PPV for abnormal scans provides insight into the number of FN scans that the CAD system falsely excludes, where nodules are present but not detected. This trade-off between scan-level specificity and sensitivity allows for the selection of an appropriate threshold depending on the clinical context, thereby reducing the physician’s burden by leveraging the evaluated performance of the CAD system primarily for screening purposes.

For example, in Figure 3a, the red dot represents the model’s performance at a cut-off threshold where the scan-level specificity is 0.80 and the scan-level sensitivity is 0.90. A specificity of 0.80 means that 80% of negative scans are correctly identified as true negatives, reducing unnecessary scan reading time and labor for 80% of normal scans during the screening stage. Conversely, a sensitivity of 0.90 indicates that 90% of positive scans are correctly identified as true positives, meaning that these scans contain nodules. However, because this is a scan-level analysis, it does not account for the number of nodules present in each true positive scan or the number of FPs. As a result, each positive scan requires a thorough review by the radiologist to accurately identify all nodules.

### 2.2. Per-Nodule Analysis

#### 2.2.1. Nodule-Level Sensitivity

Nodule-level sensitivity was calculated as the ratio of correctly identified nodules to the total number of actual nodules across the entire test set.

#### 2.2.2. Quantification

The FROC curve is the primary evaluation method used in per-nodule analysis. The horizontal axis represents the number of FPs per scan as a calculation unit, whereas the vertical axis indicates nodule-level sensitivity. Unlike the ROC curve, which evaluates performance at the scan level, the FROC curve provides a more detailed assessment of the CAD system’s ability to detect individual nodules. An example of the FROC curve is shown in Figure 3b.

Instead of mathematically integrating the area under the FROC curve, the CPM was used for quantification. The CPM represents the average sensitivity at predefined FPs per scan and provides a single numeric value ranging from zero to one. For example, if the predefined FPs per scan are set to one, two and four, and the sensitivities at these thresholds are 0.70, 0.80 and 0.90, respectively, the CPM is 0.80.

Although per-nodule analysis using the FROC curve provides valuable insights into CAD system performance, it has several limitations, as outlined below.

**Problem** **1.** *There is no standardized cut-off for evaluation, making it challenging to compare results across different studies or systems. For the LUNA16 challenge, the CPM was calculated at decision thresholds of 0.125, 0.25, 0.5, 1, 2, 4 and 8 FPs per scan. However, this is only one example that is commonly used in lung nodule detection studies for comparative purposes. If the CPM were calculated using thresholds of 0.125, 0.25, 0.5 and 1 FP per scan, the resulting CPM value would be lower. Conversely, using thresholds of one, two, four, and eight FPs per scan would result in higher CPM values. Thus, the CPM is not a standardized metric.*

**Problem** **2.** *The horizontal axis of the FROC curve represents the number of FPs that can theoretically be extended to infinity. This implies that the area under the FROC curve is not normalized and can range from zero to infinity. For example, in the Tumor-Infiltrating Lymphocytes in Breast Cancer (TIGER) challenge, 10, 20, 50, 100, 200 and 300 FPs per mm^2^ were used to evaluate lymphocyte and plasma cell detection in whole slide images. However, the specific number of FPs per scan for such calculations should be empirically determined in a data-centric manner. If the same cut-off values used in the TIGER challenge were applied to lung nodule detection, the evaluation would not be reasonable. Because of these differences, calculating the area under the FROC curve may not be an appropriate method for quantification.*

**Problem** **3.** *A case with an exceptionally high number of FPs can disproportionately affect the overall results, potentially leading to the misinterpretation of the CAD system’s effectiveness. The FROC curve was used to calculate the average FPs per scan across the entire test set. Consequently, a dataset in which 1 out of 100 scans had 100 FPs and the remaining 99 scans have none yields the same sensitivity at 1 FP per scan as a dataset where each of the 100 scans had exactly 1 FP.*

**Problem** **4.** *The CPM is highly dependent on the characteristics of the test set, which can further impact the reliability of the evaluation. Consider a CAD system with a PPV of 0.50 and a sensitivity of 0.90. In test set A, which contained 100 scans and 200 true nodules (averaging 2 nodules per scan), the CAD system detected 180 nodules (0.90 sensitivity) and generated 180 FPs, resulting in 1.8 FPs per scan. Now, consider test set B with 100 scans and 1000 true nodules (averaging 10 nodules per scan). The CAD system detected 900 nodules (0.90 sensitivity) and generated 900 FPs, yielding 9 FPs per scan. In both cases, despite having the same PPV, the number of FPs per scan varied because of the different characteristics of the test sets. Thus, even with the same CAD system, the conditions required to achieve a specific PPV can vary, depending on the characteristics of the dataset. This demonstrates that the FROC curve is heavily influenced by the number of true nodules in the test set, making it highly sensitive to the composition of the dataset.*

To the best of our knowledge, there is no established method to correct for the impact of a dataset imbalance or related issues on the FROC curve, nor are there guidelines for the composition of test datasets. Owing to the unstandardized aspects of the FROC curve and the CPM, which are commonly used in per-nodule analysis, clinical trial protocols often limit the number of nodules in the test set.

#### 2.2.3. Clinical Implication

Per-nodule analysis, which relies on the FROC curve and CPM, offers a detailed assessment of the performance of a CAD system in detecting individual nodules. This approach is especially important in clinical situations where each detected nodule must be meticulously evaluated for malignancy, such as in pre-surgical planning for patients with lung cancer. The accurate assessment of the risk of malignancy for each nodule is vital for determining an appropriate treatment strategy. Additionally, by evaluating the performance relative to the number of FPs, per-nodule analysis provides insights into the potential diagnostic workload for clinicians. This helps estimate how many FPs a physician may need to review, allowing for a better balance between the system’s sensitivity and the practical demands of the clinical workflow.

For example, in Figure 3b, the red dot represents the model’s performance at a cut-off threshold where the nodule-level sensitivity is 0.80 and the number of FPs per scan is 13. A sensitivity of 0.80 indicates that 80% of nodules present in positive scans are correctly detected, thereby reducing the labor required to meticulously review each slice to identify nodules. However, 20% of true nodules remain undetected, indicating that relying solely on the CAD system could result in missed nodules and potential risks. The 13 FPs per scan imply that to achieve this 80% detection rate, the model produces an average of 13 FPs per scan. Therefore, clinicians should not assume that all detected nodules are true positives, as each detected nodule requires manual confirmation. Nevertheless, this CAD system allows clinicians to focus on verifying detected nodules rather than manually searching across all slices, potentially reducing the overall diagnostic effort.

### 2.3. Combined Analysis

#### 2.3.1. Alternative FROC Curve

To overcome the limitations of assessing sensitivity and specificity at the case level in the ROC curve, the FROC curve was introduced to evaluate the performance at the nodule level. However, a key limitation of the FROC curve is that the horizontal axis represents the number of FPs per scan, which can theoretically range from zero to infinity, leading to a non-standardized evaluation. Therefore, the AFROC curve was proposed as an alternative to address the strengths and weaknesses of these metrics.

The AFROC curve plots the specificity from a per-scan perspective and the sensitivity from a per-nodule perspective. This means that the horizontal axis corresponds to that of the ROC curve, whereas the vertical axis aligns with that of the FROC curve. Because both axes are normalized between zero and one, the AUAFROC provides a normalized value ranging from zero to one. To plot the AFROC curve, both normal and abnormal scans must be included, similar to the per-scan analysis. An example of an AFROC curve is shown in Figure 3c.

The AFROC curve evaluates the CAD system’s performance not by focusing on the number of FPs but rather by assessing how well negative scans are correctly predicted as true negatives, thereby reducing unnecessary scan reading. At the same time, it addresses the ROC curve’s limitation of not evaluating individual nodule performance by incorporating nodule-level sensitivity. Thus, the AFROC curve provides a method to simultaneously assess the reduction in unnecessary scan reading and the system’s actual nodule detection performance.

However, there are some drawbacks. Regardless of the number of FPs present in a normal scan, only the FP with the highest probability was considered in the evaluation, whereas the others were disregarded. Additionally, the AFROC curve only evaluates nodule-level sensitivity in abnormal scans. Theoretically, even if there were an infinite number of FPs with a probability of one, this would not negatively impact the analysis using the AFROC curve.

#### 2.3.2. Clinical Implication

The AFROC curve is effective for balancing scan-level specificity and nodule-level sensitivity, making it useful in scenarios such as routine screening or lung cancer evaluations, where both the accurate detection and minimization of FPs are important. However, considering only the highest probability of FPs in normal scans can underestimate the diagnostic burden.

For example, in Figure 3c, the red dot represents the model’s performance at a cut-off threshold where the nodule-level sensitivity is 0.80 and the scan-level specificity is 0.60. As shown in Figure 3b, a sensitivity of 0.80 indicates that 80% of all nodules present in positive scans are correctly detected. Similarly, a specificity of 0.60, as seen in Figure 3a, indicates that 60% of negative scans are correctly identified as true negatives, reducing unnecessary scan reading time and labor for 60% of normal scans during the screening stage. This analysis method not only provides an assessment of how much normal scan review time could be reduced but also offers insight into the system’s overall nodule detection performance, addressing the risk of missed nodules. However, a limitation is that it does not provide information on the number of FPs included among the detected candidates.

## 3. Matching Criteria

In CAD systems, the methods used to match the output of the system with the ground truth target object can affect performance. The matching criteria may vary depending on the type of ground truth and output produced by the detection model.

### 3.1. Bounding Box-Based Method

**Figure 4:**
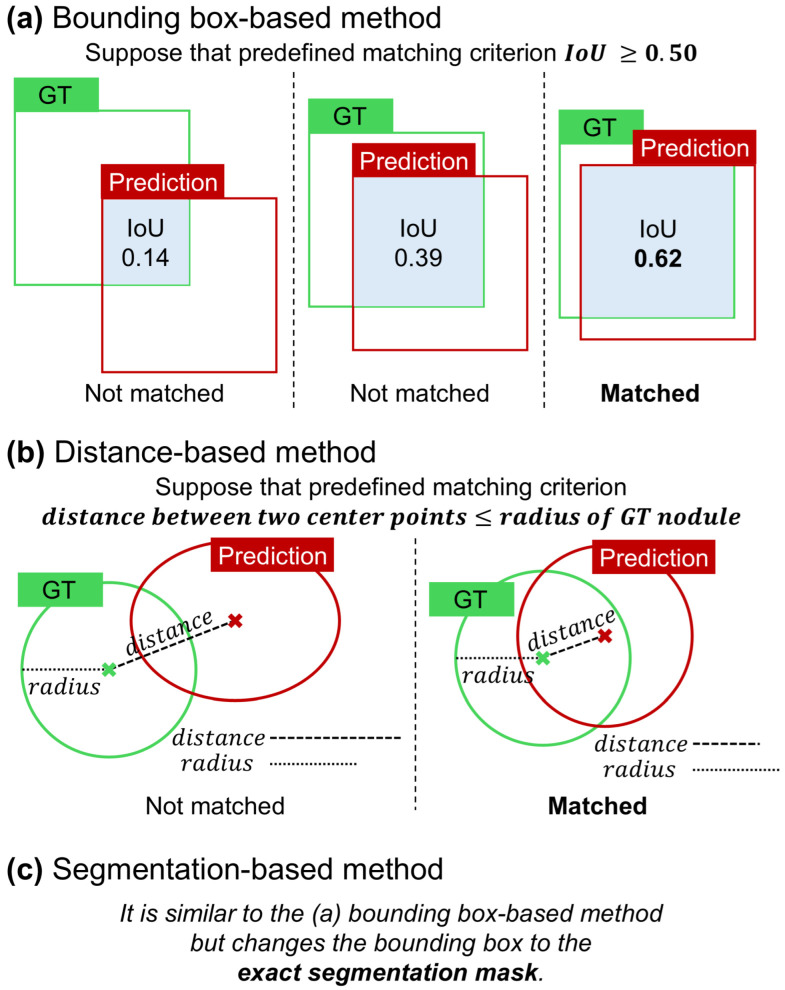
Visualization of different matching criteria used in computer-aided detection systems. (**a**) Bounding box-based method. (**b**) Distance-based method. (**c**) Segmentation-based method. IoU, intersection over union; GT, ground truth.

The bounding box-based method is one of the most widely used approaches for establishing matching criteria in the analysis of detection models [1]. As illustrated in Figure 4a, the object localization accuracy is typically assessed by calculating the intersection over union (IoU) between the predicted bounding box and ground truth. If the IoU exceeds a predefined threshold, the prediction is deemed a correct match. This method has been extensively adopted owing to its simplicity and effectiveness, particularly in scenarios in which the object’s location can be adequately represented by a rectangular region. Additionally, the bounding box approach is popular because it only requires the coordinates of the box corners, eliminating the need for more complex annotations and making it the most straightforward and cost-effective option for many applications.

However, in the context of lung nodule detection, it is crucial not only to identify the target nodule but also to analyze its various characteristics [22]. In particular, nodule size is clinically important. Consequently, CAD systems developed for lung nodule detection often employ matching methods beyond simple bounding boxes [15].

### 3.2. Distance-Based Method

This method was proposed as an alternative to the bounding box-based approach, specifically tailored to the characteristics of medical images and target objects. It is particularly effective when the ground truth is represented by the center point and the average diameter. In the case of lung nodules, this type of annotation is necessary because nodule size is clinically significant in assessing risk. In CAD systems that utilize the center point coordinates and nodule size as inputs, the output is similar in the form of the center point coordinates and nodule size, such as the radius or diameter [23]. As illustrated in Figure 4b, the matching criterion in this context is based on the Euclidean distance between the predicted center point and the ground truth center point. If this distance falls within a specific threshold, which is typically set to be less than or equal to the radius or diameter of the nodule, the prediction is considered to be a match [15].

### 3.3. Segmentation-Based Method

Because it can be difficult to fully understand the characteristics of a detected nodule based solely on the results of the detection model, some CAD systems incorporate a segmentation model to provide additional details [24,25]. In scenarios in which the CAD system produces a segmentation map as the output, such as nodule segmentation tasks in medical imaging, the matching process becomes more complex. As shown in Figure 4c, instead of using bounding boxes, the output is a mask that delineates the exact shape and area of the object. The match is determined by calculating the IoU between the predicted segmentation mask and ground truth mask. Similarly to the bounding box method, a threshold is applied to the IoU score to determine whether the segmentation result correctly identified the target object. This method offers a more detailed and precise assessment of a model’s performance, particularly in tasks where object boundaries are irregular and need to be captured accurately.

## 4. Guidelines

**Figure 5:**
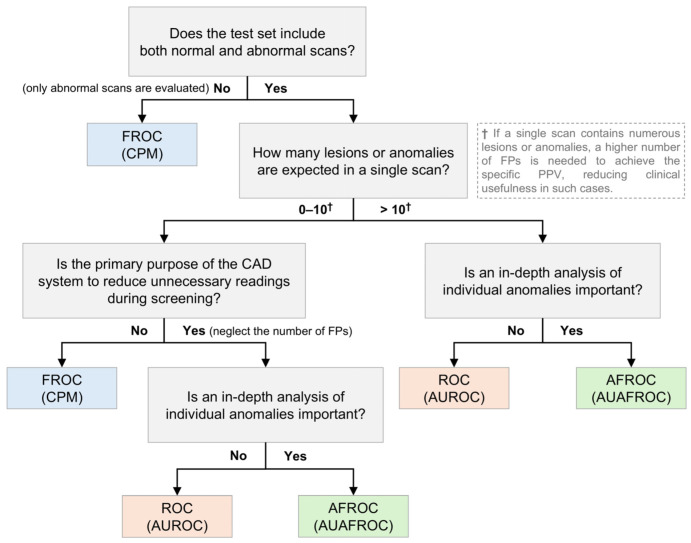
A guideline for selecting evaluation metrics for computer-aided detection systems. ROC, receiver operating characteristics; FROC, free-response ROC; AFROC, alternative FROC; CPM, competition performance metric; AUROC, area under the ROC curve; AUAFROC, area under the AFROC curve; FP, false positive; PPV, positive predictive value; CAD, computer-aided detection.

Because the characteristics of CAD systems vary based on data and specific tasks, selecting the most appropriate performance metric for each situation is challenging. Considering the strengths and limitations of individual metrics, this section aims to provide guidelines on which metrics are most appropriate for evaluating CAD systems under different circumstances, as shown in Figure 5. This serves as a reference guideline, and for a more comprehensive analysis, it is recommended to use multiple metrics in combination. Although several studies have sought to present guidelines or evaluation criteria for clinical applications from multiple perspectives, to the best of our knowledge, no study has yet proposed a gold standard for evaluation metrics [26,27].

First, it is important to establish what kind of dataset will be collected—specifically, whether the test set will evaluate normal scans and abnormal scans together. If the CAD system’s performance is assessed using only abnormal scans without normal scans, the ROC and AFROC cannot be utilized; therefore, the FROC should be employed for evaluation. Conversely, if both normal and abnormal scans are evaluated together, the ROC, FROC, and AFROC can be used as evaluation metrics.

Second, the expected number of lesions or anomalies per scan plays a significant role in the metric selection. When only a few lesions or anomalies are expected, metrics such as the FROC and CPM offer valuable insights into the sensitivity of the system and management of FPs. However, if a higher number of lesions or anomalies is anticipated, these metrics may become less reliable, potentially leading to skewed or less meaningful evaluations [21]. This is because, when there are more lesions or anomalies, achieving a certain level of the PPV requires a proportional increase in FPs. In clinical situations, having many FPs reduces clinical usefulness. For example, in the TIGER challenge, which utilizes pathological images, the lymphocyte detection task employs FROC curves with the number of FPs ranging from 10 to 300 per mm^2^ [28]. Although this high number of FPs is acceptable owing to the specific nature of pathological images, a similar abundance of FPs in general radiological images would markedly diminish the clinical utility of the CAD system. Therefore, in such cases, ROC or AFROC metrics may offer a more standardized evaluation.

Third, the specificity and negative predictive value (NPV) are critical in scenarios where the CAD system is used for routine screening, particularly if the goal is to minimize the burden of unnecessary readings or follow-ups. Metrics that emphasize the accurate identification of normal scans, such as the AUROC or AFROC, with a focus on specificity, could be useful. However, when the primary concern is the detection of abnormalities in abnormal scans, metrics such as the FROC or CPM, which emphasize sensitivity and the PPV, should be prioritized.

In summary, determining a universally optimal metric is challenging, because CAD systems and datasets vary widely. However, by carefully considering the specific task, expected scan characteristics, and clinical goals, we can guide the selection of the appropriate metrics.

## 5. Discussion

In this study, we provided a comprehensive overview of the various performance metrics and matching criteria used to evaluate CAD systems, particularly in the context of lung nodule detection in CT images. We explored the strengths and limitations of commonly used methods and metrics, such as the ROC, AUROC, FROC, CPM, AFROC and AUAFROC, as well as different matching methods, bounding box-based, distance-based, and segmentation-based, to provide a deeper understanding of how these tools can be effectively applied in clinical practice.

Although various metrics are available, a significant challenge persists because of the absence of a universally accepted ‘gold standard’ for evaluating CAD systems across various applications. The effectiveness of each metric can vary depending on the composition of the test set, characteristics of the dataset, and primary purpose of the CAD system, complicating direct comparisons of results across different studies or systems. Moreover, each metric provides different types of information. For example, although the ROC and AUROC are effective for broad scan-level assessments, they may overlook crucial details at the nodule level. In contrast, the FROC and CPM offer more detailed insights, but lack standardization and are highly sensitive to the specific composition of the test set.

This variability underscores the importance of understanding the unique properties and appropriate contexts of each metric. Without thorough knowledge of these metrics, researchers and clinicians may find it challenging to select the most suitable evaluation method for their specific needs. It is crucial to recognize that the choice of metric can significantly influence the interpretation of a CAD system’s performance and clinical utility. Therefore, the selection should be carefully aligned with the clinical objectives and characteristics of the dataset.

## 6. Conclusions

Although no single metric is universally optimal for all scenarios, a thorough understanding of available metrics, including their respective advantages and limitations, is essential. By carefully considering these factors, researchers and clinicians can ensure that they use the most appropriate tools to evaluate CAD systems under specific circumstances, leading to more reliable and clinically relevant outcomes.

## Data Availability

Not applicable.
